# fMRI Insights into Visual Cortex Dysfunction as a Biomarker for Migraine with Aura

**DOI:** 10.3390/neurolint17020015

**Published:** 2025-01-21

**Authors:** Damian Pikor, Natalia Banaszek-Hurla, Alicja Drelichowska, Mikołaj Hurla, Jolanta Dorszewska, Tomasz Wolak, Wojciech Kozubski

**Affiliations:** 1 Laboratory of Neurobiology, Department of Neurology, Poznań University of Medical Sciences, 60-355 Poznan, Poland; 2 World Hearing Center, Bioimaging Research Center of Institute of Physiology and Pathology of Hearing, 05-830 Kajetany, Poland; 3 Chair and Department of Neurology, Poznań University of Medical Sciences, 60-355 Poznan, Poland

**Keywords:** migraine with aura (MwA), visual cortex dysfunction, functional magnetic resonance imaging (fMRI), cortical hyperexcitability, neuroimaging biomarkers

## Abstract

Migraine with aura (MwA) is a common and severely disabling neurological disorder, characterised by transient yet recurrent visual disturbances, including scintillating scotomas, flickering photopsias, and complex geometric patterns. These episodic visual phenomena significantly compromise daily functioning, productivity, and overall quality of life. Despite extensive research, the underlying pathophysiological mechanisms remain only partially understood. Cortical spreading depression (CSD), a propagating wave of neuronal and glial depolarisation, has been identified as a central process in MwA. This phenomenon is triggered by ion channel dysfunction, leading to elevated intracellular calcium levels and excessive glutamate release, which contribute to widespread cortical hyperexcitability. Genetic studies, particularly involving the *CACNA gene* family, further implicate dysregulation of calcium channels in the pathogenesis of MwA. Recent advances in neuroimaging, particularly functional magnetic resonance imaging (fMRI), have provided critical insights into the neurophysiology of MwA. These results support the central role of CSD as a basic mechanism behind MwA and imply that cortical dysfunction endures beyond brief episodes, possibly due to chronic neuronal dysregulation or hyperexcitability. The visual cortex of MwA patients exhibits activation patterns in comparison to other neuroimaging studies, supporting the possibility that it is a disease-specific biomarker. Its distinctive sensory and cognitive characteristics are influenced by a complex interplay of cortical, vascular, and genetic factors, demonstrating the multifactorial nature of MwA. We now know much more about the pathophysiology of MwA thanks to the combination of molecular and genetic research with sophisticated neuroimaging techniques like arterial spin labelling (ASL) and fMRI. This review aims to synthesize current knowledge and analyse molecular and neurophysiological targets, providing a foundation for developing targeted therapies to modulate cortical excitability, restore neural network stability, and alleviate the burden of migraine with aura. The most important and impactful research in our field has been the focus of this review, which highlights important developments and their contributions to the knowledge and treatment of migraine with aura.

## 1. Introduction

MwA constitutes a highly prevalent and profoundly disabling neurological disorder, typified by transient visual disturbances such as scintillating scotomas, flickering photopsias, and intricate geometric visual patterns. These episodic phenomena, albeit temporary, exert a substantial adverse impact on daily functional capacity and occupational productivity. Despite decades of rigorous scientific inquiry, the precise pathophysiological mechanisms underpinning migraine aura remain the subject of considerable debate within the neuroscientific community. Contemporary evidence implicates a complex interplay between cortical hyperexcitability, vascular dysregulation, and aberrant neuronal signalling, which collectively underpin both the visual disturbances characteristic of aura and the subsequent headache phase [1]. A key component of this process is CSD, a wave of neuronal and glial depolarisation, which is triggered by ion channel dysfunction, elevated Ca^2+^ levels, and excessive glutamate release [2,3]. The advent of advanced neuroimaging modalities, particularly fMRI, has heralded a paradigm shift in elucidating cortical function within the context of migraine pathophysiology. By capturing dynamic alterations in cerebral blood oxygenation levels, fMRI affords critical insights into the temporal and spatial patterns of neural activity within the visual cortex during distinct phases of the migraine cycle [4]. Notably, empirical investigations have consistently demonstrated pronounced hyperactivation of the visual cortex during aura episodes, succeeded by a state of cortical hypoactivity in the interictal phase. This cyclical modulation of cortical activity provides robust support for the hypothesis that cortical spreading depression constitutes a core mechanistic driver of migraine aura. Furthermore, this evidence underscores the persistence of cortical dysfunction beyond the episodic clinical manifestations of the disorder, thereby suggesting a chronic neurophysiological underpinning [5,6]. Complementary findings derived from fMRI studies have revealed enduring abnormalities in baseline cortical activity among individuals with MwA, even in the absence of overt aura symptoms [7]. These observations suggest that the visual cortex in individuals with MwA remains in a state of heightened excitability or functional dysregulation, potentially predisposing these individuals to recurrent episodic attacks [8,9]. Comparative analyses have further delineated that the cortical activation patterns observed in MwA are markedly distinct from those associated with other primary headache disorders in the visual cortex, demonstrating substantially different activation profiles [10]. These differentiating characteristics underscore the potential utility of visual cortical dysfunction as a disease-specific biomarker for MwA. The specificity of visual cortical abnormalities in MwA is further corroborated by neuroimaging investigations demonstrating increased cortical activation during aura episodes, a phenomenon absent in patients experiencing non-migrainous headache disorders. For instance, studies employing resting-state fMRI have identified augmented functional connectivity and excitability within the visual and pain-processing neural networks of individuals with MwA, relative to healthy controls [11]. These findings align with prevailing theories of cortical hyperexcitability and suggest a broader dysregulation of neuronal networks integral to sensory integration and processing [12,13,14]. Collectively, these insights not only enhance the understanding of the complex pathophysiological substrates underpinning MwA but also highlight potential avenues for the development of targeted therapeutic strategies aimed at mitigating cortical excitability and restoring network homeostasis.

## 2. Pathophysiology of Migraine with Aura

**Figure 1:**
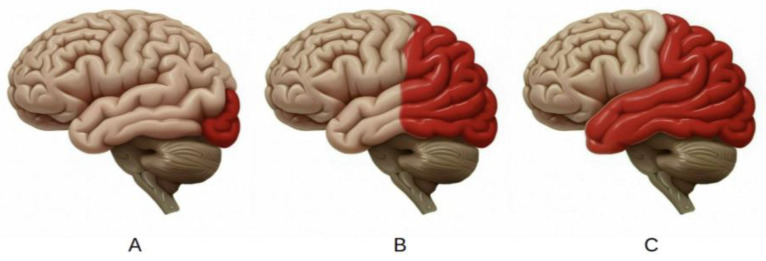
(**A**–**C**) Cortical spreading depression causes migraine aura and consists of a wave of depolarization of allcortical elements that spreads at rate of 2–6 mm per’min (red).

Migraine with aura is a neurological condition characterized by cortical spreading depression (CSD), a propagating wave of neuronal depolarization followed by inhibition. This disruption in cortical function produces aura symptoms such as visual or sensory disturbances. CSD propagates across the cortex at a rate of 2–6 mm/min, resulting in the characteristic sensory disruptions [15,16], [Figure 1]. While vascular changes such as vasoconstriction and vasodilation were once thought to be primary drivers of migraines, current evidence indicates they are secondary to neuronal activity and not essential for the therapeutic efficacy of antimigraine agents [17,18]. Genetic predispositions, including rare monogenic variants, contribute to CSD susceptibility, alongside environmental and physiological triggers. Additionally, comorbidities such as depression, epilepsy, and stroke share overlapping mechanisms with CSD, underscoring its central role in migraine pathophysiology [19].

### 2.1. CACNA Genes in Migraine with Aura

The pathophysiology of migraine aura is intrinsically connected to genetic factors, particularly mutations in the *CACNA1A gene*, which encodes the alpha-1 subunit of the CaV2.1 calcium channel. This channel is integral to the regulation of calcium ion influx within neurons, a process that is essential for synaptic transmission and neuronal excitability. Mutations in *CACNA1A* are directly implicated in familial hemiplegic migraine type 1 (FHM1), a rare autosomal dominant subtype of migraine. These mutations enhance the excitability of CaV2.1 channels by reducing their activation threshold, thereby increasing calcium influx into presynaptic terminals. The resultant elevation in calcium influx promotes excessive glutamate release, precipitating CSD, a fundamental mechanism underlying the transient visual disturbances characteristic of migraine aura [20,21,22,23]. Notable mutations, including T666M, R192Q, and S218L, further intensify neuronal hyperexcitability, facilitating the propagation of CSD and contributing to the protraction of aura symptoms [24,25]. Beyond *CACNA1A*, other genes within the *CACNA* family, such as *CACNA1B* (encoding CaV2.2) and *CACNA1E* (encoding CaV2.3), have also been implicated in the pathogenesis of migraine aura. For example, mutations in *CACNA1B* have been identified in families affected by migraine, while a polymorphism in *CACNA1E* (rs35737760) has been associated with patients exhibiting complex neurological aura phenotypes [26,27]. These genetic variations contribute to perturbations in calcium signalling and heightened neuronal excitability, both of which are critical for the initiation and propagation of CSD. Moreover, the presence of antibodies targeting *CACNA1H* has been documented in individuals with HaNDL (headache attributed to neurological deficits and cerebrospinal fluid lymphocytosis), a rare headache syndrome, suggesting an immunological dimension to the involvement of *CACNA* genes in aura-related disorders [28]. Collectively, these findings underscore the pivotal role of *CACNA* gene family dysregulation in the intricate pathophysiology of migraine aura and its diverse clinical manifestations.

### 2.2. Vascular Dysregulation and Migraine Aura

CSD is recognised as the primary mechanism underlying migraine aura and may also trigger trigeminal sensory activation, which contributes to the headache phase. This challenges earlier theories that emphasised vasodilation as the central pathophysiological process in migraine. It is now understood that vasodilation is secondary, and vasoconstriction is no longer considered crucial for antimigraine efficacy. Instead, the key event is a wave of neuronal and glial depolarisation spreading across the cortex, causing transient cortical hyperperfusion followed by hypoperfusion, as documented through perfusion-weighted MRI imaging [29]. Studies of fremanezumab have shown its ability to reduce CSD propagation velocity, although it does not fully prevent it. During the aura, cerebral blood flow (CBF) initially increases, reflecting neuronal excitation, before decreasing during the hypoperfusion phase as neuronal activity diminishes. Calcium signalling plays a central role in this vascular response, with calcium influx into vascular smooth muscle cells regulating vasoconstriction and vasodilation. Calcium channel blockers (Ca^2+^ blockers), such as flunarizine, reduce these effects by inhibiting calcium influx, promoting vasodilation, and stabilising CBF, potentially alleviating the hypoperfusion phase and reducing the likelihood of headache progression [30]. Imaging and cortical morphological studies reveal that these vascular changes are restricted to the aura phase and do not extend to the headache phase, supporting the idea that the aura and headache phases are governed by distinct mechanisms [31]. Surface-based morphometric studies also identify structural alterations in cortical regions related to pain processing in chronic migraine patients [32]. This growing understanding has led to more targeted therapies. Acute treatments commonly involve NSAIDs, simple analgesics, and triptans (5HT1B/1D receptor agonists), while ergot derivatives have declined due to cardiovascular safety concerns. New therapies, including CGRP receptor antagonists (gepants) and lasmiditan (a selective 5HT1F receptor agonist), provide effective alternatives for acute migraine management. Preventative strategies, including Ca^2+^ blockers, stabilise vascular tone and may offer neuroprotective benefits by modulating intracellular calcium levels, reducing neuronal hyperexcitability, and attenuating the CSD wave [33].

### 2.3. Neuronal Hyperexcitability and Sensory Dysregulation

Migraine with aura is characterised by a complex pathophysiological mechanism, one of the key components of which is cortical hyperexcitability. Neurophysiological studies show a marked increase in cortical reactivity, particularly in response to sensory stimuli. Visual evoked potentials (VEPs) in patients with migraine with aura demonstrate elevated amplitudes and altered temporal patterns, indicating significant disturbances in neuronal processes. This phenomenon may be a result of dysfunction in the neuronal systems responsible for regulating cortical excitability [34]. A central aspect of the pathophysiology of migraine with aura is the disruption of the balance between excitatory and inhibitory neurotransmitters. Glutamate, the main excitatory neurotransmitter, is frequently overactivated in patients with migraine with aura, leading to excitotoxicity and subsequent neuronal dysfunction. At the same time, decreased GABA (gamma-aminobutyric acid) activity, the principal inhibitory neurotransmitter, may contribute to the lack of effective modulation of neuronal excitability. This imbalance affects the processes of neuronal depolarisation and repolarisation, potentially promoting the development of CSD, a key mechanism associated with the migraine aura [35]. Patients with migraine with aura show abnormal processing of sensory stimuli, particularly in visual regions. Functional imaging studies, such as fMRI, reveal dysfunction in visual cortex activation during the perception of visual stimuli [36]. The abnormal neuronal response may arise from cortical hyperexcitability and weakened GABAergic inhibition, leading to the characteristic patterns observed during the aura phase. Furthermore, sensory dysfunction extends beyond the visual cortex, influencing other brain regions, including the somatosensory cortex and areas involved in auditory and olfactory processing. Dysfunctional activity in the visual cortex of patients with migraine with aura is not confined solely to the attack periods. Research indicates persistent alterations in visual cortical function between attacks, suggesting a chronic state of neuronal dysregulation. Persistent hyperexcitability and reduced synaptic plasticity may impact patients’ everyday functioning, leading to sensory hypersensitivity and impaired perceptual abilities [37].

### 2.4. Neurovascular Coupling and Aura Pathogenesis

**Figure 2:**
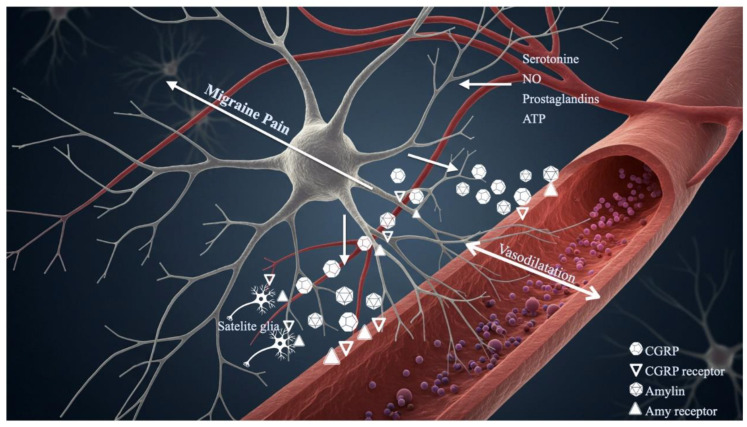
This representation illustrates the key components of the trigeminovascular system, comprising meningeal blood vesselsand trigeminal nerve fibres, which are actively involved during migraine headache attacks. The binding of calcitoningene-related peptide (CGRP) and amylin to their respective receptors induces vasodilation within meningeal vesselsFurthermore, CGRP activates mast cells, prompting the release of numerous pro-nociceptive mediators, ineludingserotonin, adenosine triphosphate (ATP), prostaglandins, and nitric oxide (NO), These mediators further excitenociceptive fibres, leading to the amplification of CGRP release and the enhancement of pain signalling pathwaysassociated with migraines.

A key factor in the pathogenesis of migraine aura is neurovascular coupling, the physiological mechanism that combines cerebral blood flow with neuronal activity. One of the main mechanisms in the formation of auras is cortical spreading depression CSD, which is characterized as a brief propagating wave of neuronal depolarization followed by a prolonged interval of inhibition. The onset of aura symptoms is correlated with the onset of an initial period of regional hyperaemia, which is followed by a prolonged oligemia [29,38]. Sophisticated neuroimaging modalities, including fMRI, ASL, and positron emission tomography (PET), have elucidated critical haemodynamic changes, delineating their spatiotemporal relationships with cortical and subcortical structures, such as the thalamus and brainstem [39]. CSD also initiates mechanisms of peripheral and central sensitisation that contribute to the manifestation of migraine headaches [40]. Experimental models have demonstrated that pharmacological agents, such as calcitonin gene-related peptide (CGRP) receptor antagonists, which attenuate CSD susceptibility, effectively mitigate migraine attacks [41]. Additionally, genetic studies in murine models have provided compelling evidence of the influence of migraine-associated genetic variants on CSD dynamics, thereby reinforcing the link between genetic predisposition and neurovascular dysfunction [42,43]. Vasoactive peptides modulate cerebrovascular flow and interact with neuronal and glial components, exacerbating both aura and headache symptomatology [44]. Furthermore, the neuroendocrine hormone amylin is essential for nociceptive signalling and the pathogenesis of migraines. In preclinical studies, it has been demonstrated to cause orofacial allodynia and increase neuronal activity within the trigeminovascular system (TVS) [45,46], captivatingly 41% of migraineurs have been shown to experience migraine-like episodes after using pramlintide, an amylin analogue [45]. The Amylin 1 (AMY1) receptor, which is mostly expressed in C-fibre neurons of the trigeminal ganglion, mediates the actions of amylin inside the TVS [47]. Interestingly, CGRP is also bound by the AMY1 receptor, which allows CGRP to function across a variety of signalling pathways [48], [Figure 2]. The presence of many CTR splice variants is thought to be responsible for the pharmacological complexity of the AMY1 receptor, which forms a heterodimeric complex with the calcitonin receptor (CTR) and receptor activity-modifying protein 1 (RAMP1). When the AMY1 receptor is activated in migraines, either cyclic adenosine monophosphate (cAMP) builds up or protein kinase C (PKC) is activated, which then blocks large-conductance calcium-activated potassium (BKCa) channels [48,49]. Examining the functional functions and variations in expression of these receptor isoforms may help create tailored treatments that reduce off-target effects and lessen migraines. Another neuropeptide in the calcitonin family, PACAP, is crucial to the pathophysiology of migraines. Between 58 and 72% of migraineurs experience migraine-like episodes after receiving PACAP peptide infusion [50,51]. The three G protein-coupled receptors (GPCRs) that PACAP acts through are PAC1, VPAC1, and VPAC2. At the PAC1 receptor, PACAP has noticeably higher activity than vasoactive intestinal peptide (VIP) [52]. Both the cAMP and PKC signalling pathways are activated by PAC1 receptor activation through alternative splicing, with cAMP being the major route [53,54]. In order to develop focused, effective treatments with reduced off-target action and enable customized therapy paradigms, a thorough understanding of receptor variant expression patterns is essential. A key modulator of vascular tone, nitric oxide (NO), is closely linked to the pathophysiology of migraines. Vasodilation and neuropeptide release result from NO diffusing into cellular compartments and activating soluble guanylate cyclase, which raises intracellular cyclic guanosine monophosphate (cGMP) levels [55]. In susceptible people, glyceryl trinitrate, a precursor of NO, consistently causes migraine-like headaches. On the other hand, it has been demonstrated that blocking NO synthases reduces migraine attacks [56]. Furthermore, drugs including prostacyclin, m-chlorophenylpiperazine, and reserpine trigger migraine attacks by activating NO synthases [55]. These results highlight the crucial part that NO plays in the aetiology of migraines and make it an attractive target for therapeutic intervention in migraine treatment.

## 3. fMRI Insights and Visual Cortex Dysfunction

fMRI relies on the blood oxygen level-dependent (BOLD) contrast, which detects changes in regional blood flow and oxygenation associated with neuronal activity. This non-invasive technique measures signal variations caused by magnetic properties of deoxygenated haemoglobin, enabling the localisation of functional brain regions during specific cognitive or motor tasks.

### 3.1. Methodology in Migraine Studies

fMRI has become an irreplaceable instrument in gathering details about human brain connectivity and interactions, not only in healthy subjects but also in patients suffering from a myriad of diseases. Utilising BOLD signals, fMRI allows investigators to asses neuronal functioning and coactivation, giving a broader understanding of processes involved in sensory and cognitive functioning [57,58]. Such methods integrate neuronal activity, cell metabolism and local blood circulation to obtain structural maps of activation in specific brain areas, especially in rest and during task-based activities. The default mode network (DMN), salience network, and sensorimotor networks—all of which are essential for preserving cognitive and sensory homeostasis—have been mapped in neuroscience thanks in large part to fMRI. [59]. fMRI has advanced migraine studies significantly, allowing us to better understand the complex mechanisms involved in the pathology of this disease. As more and more studies appear, migraine might be seen as a network-grade disorder involving destabilization in functional and structural interconnections. A crucial method for detecting changes in brain activity is resting-state fMRI (rs-fMRI). This technique can detect disturbances in important networks such as the DMN’s decreased connectivity, the salience network’s increased activity, and the pain-processing network [60]. The brainstem, thalamus, and cortical areas, such as the insula and somatosensory cortex, may take a crucial role in the pathogenesis of migraine [61]. On the other hand, task-based fMRI (tb-fMRI) has given insight into brain reactions in response to pain stimuli or migraine triggers. For example, studies using painful thermal, visual, or auditory stimuli have revealed heightened cortical excitability and increased activation of pain-related regions during migraine attacks [62]. Furthermore, longitudinal fMRI investigations have provided evidence of maladaptive neuroplasticity, suggesting that repeated migraine attacks may induce long-term changes in pain-processing networks, contributing to chronic migraine development. Advancements in analytical techniques, such as seed-based functional connectivity analysis, independent component analysis (ICA), and graph theoretical approaches, have allowed researchers to move beyond regional activity to explore the intricate patterns of connectivity and network organization [63]. These methods have highlighted the role of hubs within the migraine brain, showing altered network efficiency and disrupted integration between pain-related and cognitive control networks. Emerging findings from fMRI studies also point to a central role of the hypothalamus and brainstem in modulating premonitory symptoms and attack initiation, further enriching the mechanistic understanding of migraine [62]. Together, these fMRI-based methodologies provide a powerful framework for studying migraine, offering not only a deeper understanding of the disrupted neurobiology but also potential biomarkers for disease progression and therapeutic response. The integration of fMRI with other modalities, such as magnetic resonance spectroscopy (MRS) or diffusion tensor imaging (DTI), holds promise for a more comprehensive exploration of structural–functional coupling in migraine, paving the way for innovative, targeted interventions.

### 3.2. Interictal Abnormalities in Visual and Non-Visual Cortices

Understanding abnormalities in the visual and non-visual cortices is a complex issue in migraine research. Many studies have focused on how cortical activity responds to painful stimulation [64]. However, subtle structural changes and interictal dysfunction in the visual cortex could reflect adaptive plasticity linked to recurrent migraine episodes, or possibly changes in blood flow or metabolism. Another perspective suggests that migraine and abnormal sensory processing share a common origin, likely influenced by hereditary factors. These shared mechanisms might manifest as increased cortical thickness or atypical interictal activity in sensory processing regions. Interestingly, research has highlighted abnormal activity in the striate and extrastriate areas during motor tasks driven by visual stimulation in migraine patients. For instance, when individuals with migraine with aura were shown incongruent lines in an fMRI block paradigm, they exhibited increased activity in the peristriate visual cortex [65]. Similarly, interictal MRI scans have revealed heightened BOLD responses in the symptomatic hemispheres following visual stimulation in those with fixed-side migraine aura [66]. Other studies point to higher activation in the contralateral sensorimotor cortex of patients with migraine without aura compared to healthy controls. This variation may stem from differences in the design of the experiments, such as the use of block paradigms in hand motor tasks [67]. Importantly, evidence from fMRI connectivity studies suggests that migraines may involve a broader interictal network disorder rather than isolated dysfunction in primary or secondary sensory areas. This complexity means terms like “increased” or “decreased” activation of visual areas oversimplify the diverse mechanisms at play in migraine-affected brains [64,68,69,70,71]. The work of Conforto et al. further supports the idea of abnormal interictal visual function in migraine, using a non-painful motor task triggered by visual stimulation to demonstrate these disruptions [72]. Moreover, abnormal processing in sensory areas, including the visual cortex, might activate the so-called “pain matrix.” This network includes the thalamus, insula, anterior cingulate cortex, prefrontal cortex, primary and secondary sensory cortices, and cerebellum. Abnormal cortical excitability is believed to be one of the key factors contributing to the onset of migraine attacks [73].

### 3.3. Visual Stimuli

In fMRI studies, one of the first changes observed in patients was the alteration of the BOLD signal. This was characterized by an increase in the mean MR signal and a reduction in stimulus-driven modulation. These changes are thought to result from elevated blood flow and blood volume, likely caused by stronger neuronal activity. In the patient group, this activity was associated with visual auras described as shining, scintillating, and migrating phenomena [74]. The exact mechanisms behind these visual phenomena are still under investigation. It remains uncertain whether any specific region within the occipital lobe initiates the spreading event. Some researchers have focused on the primary visual cortex (V1) as a potential origin [75,76], particularly because of its retinotopic organization and sensitivity to oriented inputs. This aligns with the idea that the visual representation itself is highly retinotopic and orientation-specific [77]. However, the occipital cortex is made up of numerous cortical areas, many of which, like V1, are also selective for orientation. There are various types of migraine auras. For example, auras featuring corrugated, continuous lines (often referred to as the “fortress illusion”) might originate in the V1 area. Meanwhile, auras with coloured phosphenes are thought to begin in the V8 area, known for its role in colour perception [74]. Additionally, somatosensory auras are believed to be triggered by activity in the somatosensory cortex. Interestingly, visual activation through visual stimulation is considered one of the most direct paradigms in fMRI studies. It was also among the first methods used to explore these phenomena [78].

### 3.4. Cortical Connectivity and Network Dynamics in MwA

Recent studies have provided important insights into the role of cortical connectivity and network dynamics in the pathophysiology of MwA. Functional neuroimaging and electrophysiological research consistently highlight specific alterations in visual networks that distinguish patients with MwA from those with migraine without aura (MwoA) [5,79,80]. One of the key findings is the increased functional connectivity observed in the right lateral geniculate nucleus (LG), a critical structure in the extrastriate cortex that is involved in higher-order visual processing and sensory integration [81]. This enhanced connectivity occurs even in the absence of detectable structural or microstructural abnormalities, as shown by negative results from voxel-based morphometry (VBM) and diffusion tensor imaging (DTI) studies [82]. These results suggest that the functional changes seen in MwA are not due to structural damage but rather reflect alterations in the activity of the visual pathways themselves. Electrophysiological studies, particularly those involving VEPs, have revealed that individuals with MwA exhibit abnormalities in visual information processing, including deficits in mechanisms like habituation and sensitization [83,84]. Additionally, resting-state fMRI (RS-fMRI) studies have shown an increase in functional connectivity within the resting-state visual network (RS-VN), which includes regions such as the retinotopic occipital cortex and temporo-occipital areas [85]. This increased connectivity corresponds with heightened cortical excitability and greater metabolic activity observed in the visual cortex of MwA patients. Interestingly, during the interictal period, imaging studies have demonstrated that the LG exhibits increased activity in response to both high and low luminous stimulation, which suggests a hypersensitive visual system [9,86]. This hypersensitivity could help explain why bright or flickering lights are commonly reported as triggers for migraine attacks in patients with MwA [87]. The elevated functional connectivity in the RS-VN likely requires substantial metabolic resources, providing a possible explanation for the increased energy demands observed in these patients compared to healthy controls and individuals with MwoA [88]. Importantly, the observed changes in functional connectivity are not associated with clinical features such as attack frequency, duration, or disease history [5]. This suggests that these connectivity changes are primarily related to the visual aura phenomenon itself, rather than being influenced by the overall severity of the migraine. These alterations may reflect experience-dependent plasticity within visual pathways, potentially involving changes in glutamatergic neurotransmission and synaptic remodelling [89]. In summary, patients with MwA show distinctive patterns of functional connectivity within the visual cortex, which are consistent with the unique pathophysiology of the condition when compared to MwoA. Further research employing advanced imaging techniques is needed to deepen our understanding of the underlying mechanisms of aura initiation and propagation, which could lead to more targeted treatments for this debilitating condition [90].

### 3.5. Functional Connectivity in Migraine

By analysing several studies, it is possible to identify various brain regions where functional connectivity is altered in migraine patients compared to healthy controls, particularly in areas related to pain processing, cognitive regulation, and sensory perception [91]. It is observed to reduce rs-FC in key areas of the prefrontal cortex, including the left middle frontal gyrus, superior frontal gyrus, and the left inferior triangular part of the inferior frontal gyrus. These regions are involved in pain regulation and cognitive processing. Previous research has shown that reduced connectivity in these areas correlates with increased pain intensity during migraine attacks [92]. Structural changes, such as decreased grey matter volume in the middle frontal gyrus, further support the role of these regions in migraine [93]. The left inferior frontal gyrus is particularly important for the cognitive modulation of pain and emotional regulation, and alterations here are linked to changes in pain perception in migraine sufferers [94]. Additionally, abnormalities were observed in the occipital lobe, including the calcarine, superior occipital, and middle occipital gyrus, regions critical for visual processing. These areas are associated with cortical spreading depression, a key mechanism in migraine with aura, and increased excitability in the occipital cortex has been suggested to lower the threshold for migraine attacks [74]. Furthermore, altered connectivity in the cuneus and lingual gyrus is involved in visual processing and photophobia in migraine patients [87,95]. Moreover, enhanced connectivity was observed in regions such as the right posterior cingulate and parahippocampal gyrus, which are involved in pain modulation, as well as the postcentral and precentral gyri, linked to the trigeminal–thalamo–cortical pain pathway [96]. Overall, these findings highlight widespread alterations in brain connectivity in migraine, pointing to complex mechanisms that need further exploration, particularly across different migraine subtypes and clinical profiles.

### 3.6. Comparison of fMRI Findings in MwA and Other Primary Headaches

**Figure 3:**
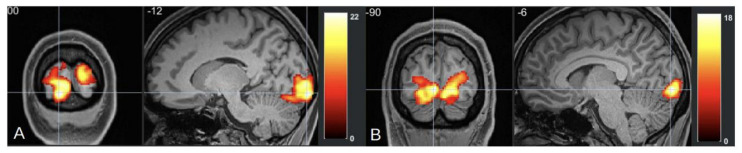
(**A**) fMRl imaging of occipital cortex of patient with migraine during aura episode, cluster size- 8068voxels, (**B**) fMRl imaging of occipital cortex of patient with tension type headaches, cluster size- 2595 voxels.

**Figure 4:**
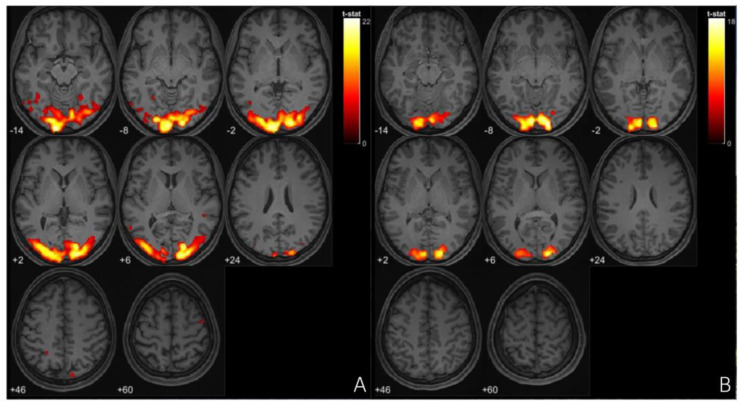
(**A**) fMRl imaging of occipital cortex of patient with migraine during aura episode, (**B**) fRl imaging ofoccipital cortex of patient with tension type headaches. In this study, both patients were presented withidentical visual stimuli during the fMRI session.

Functional MRI (fMRI) studies of migraine with aura (MA) have identified distinct neural alterations, particularly in resting-state functional connectivity (rs-FC). Increased rs-FC is observed in regions such as the occipital cortex, sensorimotor networks, and visual areas like the lingual gyrus, which play a critical role during both ictal and interictal phases of MA [97,98], [Figure 3 and Figure 4]. Altered connectivity is also found in the thalamus, insula, and cingulate cortex, suggesting the involvement of key pain-processing networks [99]. Furthermore, differences in rs-FC between MA and migraine without aura (MwoA) point to potential biomarkers distinguishing these subtypes [100]. These findings support the notion that distinct neural circuits may underlie the different clinical presentations of migraine, offering new insights into the pathophysiology of the disorder. In tension-type headache (TTH), fMRI studies highlight distinct neural changes that may offer insight into the underlying mechanisms of the condition. Increased fractional amplitude of low-frequency fluctuations (fALFF) is observed in the right posterior and anterior insula in episodic TTH patients compared to healthy controls, while reduced fALFF is found in the posterior cingulate cortex [101]. ReHo analysis reveals increased activity in the right medial superior frontal gyrus [102], though other studies report decreased ReHo in regions such as the bilateral caudate nucleus and frontal gyri [103]. These findings suggest a disrupted balance of activity across key pain and sensory processing regions, providing a better understanding of TTH’s neural basis and potential therapeutic targets. In trigeminal autonomic cephalalgia (TAC), including cluster headache (CH), fMRI studies have documented altered functional connectivity and brain activation patterns. In CH, increased rs-FC is noted between the hypothalamus and regions such as the pregenual anterior cingulate cortex, visual cortex, thalamus, and right insula [104]. Decreased FC is observed in the right frontal pole and right amygdala in chronic CH patients [105]. These altered connectivity patterns suggest that disruptions in both the sensory and emotional processing circuits contribute to the intensity of pain in CH. Additionally, TAC subtypes like short-lasting unilateral neuralgiform headache attacks with conjunctival injection and tearing (SUNCT) exhibit activation in the posterior hypothalamus, brainstem, and frontal cortices during attacks [106,107,108]. This indicates a significant involvement of the hypothalamus and brainstem in the pathophysiology of TAC. In the case of other primary headaches, such as new daily persistent headache (NDPH), fMRI studies have shown abnormal functional connectivity in brain regions involved in emotion and pain regulation [109]. These abnormalities suggest that NDPH may involve disrupted integration between pain processing and emotional regulation networks. Additionally, structural changes, including alterations in cortical area and grey matter volume, have been reported in NDPH patients [110]. These structural findings further emphasize the chronic nature of NDPH and its potential link to persistent alterations in brain structure and function. These findings underscore the complex neural mechanisms underlying these various headache disorders, suggesting that personalized treatment strategies targeting these neural alterations may hold promise for more effective management.

### 3.7. Imaging Patterns of Visual Cortex Dysfunction

fMRI has emerged as a critical tool for exploring the role of the visual cortex in MwA. It provides detailed insights into the dynamic changes occurring within this brain region during both ictal and interictal phases. The BOLD signal, which reflects variations in blood oxygenation associated with neuronal activity, has been instrumental in uncovering the functional alterations characteristic of migraine aura. Studies employing fMRI have consistently highlighted significant changes in the occipital cortex that correlate with the onset and progression of visual aura in MwA [111]. At the onset of visual aura, an increase in the BOLD signal within the occipital cortex indicates heightened neuronal activity [112]. This activation becomes more pronounced as the aura advances. Early BOLD changes occur in cortical regions aligned with the initial visual field disturbances, with further activation mirroring the aura’s expansion across the visual fields. Precise retinotopic mapping offers a robust explanation for the spatial and temporal features of migraine aura [113]. In addition to these cortical activations, fMRI studies have revealed altered connectivity between the occipital cortex and other brain regions during migraine attacks. These changes, predominantly observed in the ictal phase, are believed to contribute to the multisensory nature of migraines. For instance, functional connectivity alterations between the visual cortex and areas such as the pons, somatosensory cortex, and thalamus have been identified [114]. Such disruptions may explain the sensory hypersensitivity, allodynia, and accompanying headache pain often reported during aura. Interactions between the visual and somatosensory cortices are particularly implicated in these overlapping sensory experiences. Resting-state fMRI has further demonstrated abnormal network activity in the visual cortex of individuals with MwA, even in the absence of active migraine episodes. These disruptions in baseline connectivity suggest a predisposition to migraine attacks, potentially linked to increased sensitivity to sensory stimuli, such as light [34]. Aberrant connectivity in the visual cortex is indicative of wider disruptions in brain network activity, which may contribute to the initiation of migraines. Recent investigations have examined low-frequency BOLD signal fluctuations within the visual cortex during resting states [115]. These fluctuations, indicative of intrinsic neural activity, display altered patterns in individuals with migraine. Such irregularities are posited to result from disruptions in neurovascular coupling, which modulates cerebral blood flow in response to neuronal activity. These abnormalities may elucidate the atypical functional activity observed in the visual cortex, particularly during aura episodes. Altered low-frequency fluctuations are also likely contributors to the changes in connectivity observed during both ictal and interictal phases observed also in patients suffering from migraine without aura [93,116]. Furthermore, studies using visual stimuli in fMRI have demonstrated increased cortical excitability in the visual cortex of migraineurs. This heightened responsiveness may underlie photophobia and the rapid onset of visual aura. Research combining VEPs with fMRI has revealed exaggerated cortical responses to visual stimuli in individuals with MwA, which may account for the intensity and rapid progression of visual disturbances during aura [117]. Complementary evidence from ASL perfusion imaging has provided further insights into haemodynamic changes in the occipital cortex during migraine aura. ASL, which measures CBF, has shown transient hypoperfusion in the occipital cortex during the early stages of aura, followed by hyperperfusion. In a study of paediatric patients with MwA, ASL detected hypoperfusion in 94% of cases during aura, with concurrent vasospasm identified in 75% of cases via time-of-flight magnetic resonance angiography. These perfusion changes were closely aligned with aura lateralisation in 93% of patients, reinforcing the link between altered cerebral blood flow and the visual disturbances characteristic of migraine aura [39]. In conclusion, fMRI has significantly advanced our understanding of the functional dynamics of the visual cortex in MwA, revealing its critical role in the onset, progression, and spread of visual aura. Altered functional connectivity between the visual cortex and other brain regions contributes to the multisensory symptoms of migraines, while disruptions in resting-state activity may predispose individuals to attacks. Furthermore, changes in low-frequency BOLD fluctuations underscore the complex neural processes underlying migraine. ASL perfusion imaging corroborates these findings, highlighting cerebral blood flow alterations in the occipital cortex. Together, these studies provide a comprehensive understanding of the neurobiological mechanisms driving MwA and underscore the value of advanced imaging techniques in elucidating the pathophysiology of this condition. Ongoing research will refine these insights and inform the development of more effective diagnostic and therapeutic strategies for MwA.

## 4. Conclusions and Future Research

fMRI has emerged as a valuable biomarker for assessing visual cortex dysfunction in individuals with MwA. By utilising BOLD signals, fMRI has elucidated critical alterations in occipital cortex activity, both during ictal and interictal phases. These findings highlight the visual cortex as a central hub in the onset and progression of visual aura, driven by aberrant neurovascular coupling and increased cortical excitability. Moreover, disrupted connectivity between the visual cortex and regions such as the thalamus, pons, and somatosensory cortex underscores the multisensory nature of migraines and their associated symptoms, including photophobia and sensory hypersensitivity. Additionally, baseline network abnormalities have been found in resting-state fMRI studies in people with MwA, indicating a tendency toward elevated sensory responses. Furthermore, these findings are supported by sophisticated imaging methods such ASL perfusion imaging, which shows anomalies in cerebral blood flow, including temporary hypoperfusion and hyperperfusion in the occipital cortex during aura episodes. These vascular alterations offer strong proof that the visual abnormalities typical of MwA are caused by a combination of neuronal and hemodynamic processes. Our knowledge of visual cortical dysfunction in migraine should be further developed in future studies, paying special attention to possible interactions with the optic nerve. By examining the role that optic nerve dynamics play in aura genesis and propagation, new opportunities for early identification and tailored therapies may become available. Furthermore, a thorough framework for researching cortical excitability and vascular alterations in MwA may be developed by combining multimodal imaging techniques such as fMRI, ASL, and visual evoked potentials. Improved clinical results for migraine sufferers will result from these developments, which will guide the creation of focused diagnostic instruments and treatment plans. However, while current methodologies offer valuable insights, there are a number of limitations to consider. Despite being very informative, fMRI’s limited temporal resolution makes it difficult to record the quick onset of cortical changes during migraine episodes. Furthermore, the interpretation of neuronal activity may become more difficult due to the influence of variables like vascular fluctuations on the reliance on BOLD signals. Additionally, individual variability and the difficulty of standardizing procedures across diverse populations pose problems for other research methods, such as ASL and visual evoked potentials, which could compromise the reproducibility and generalizability of results. The studies reviewed in this manuscript have significantly advanced our understanding of the complex pathophysiology of MwA. Neuroimaging research, particularly through fMRI, has mapped dynamic alterations in the visual cortex, revealing both hyperexcitability and disrupted connectivity during ictal and interictal phases. These findings underscore the central role of CSD in aura generation and demonstrate how persistent neuronal dysregulation may predispose individuals to recurrent migraine attacks. Additionally, comparative analyses have distinguished MwA-specific cortical patterns from other headache disorders, emphasizing the potential for visual cortex dysfunction as a diagnostic biomarker. Genetic studies have further elucidated the influence of calcium channel dysregulation in the pathogenesis of MwA, offering insights into targeted therapeutic strategies.

## Data Availability

No new data were created or analyzed in this study. Data sharing is not applicable to this article.
